# Progress in Surgery

**Published:** 1900-09-22

**Authors:** 


### Progress in Surgery.

### GENERAL SURGERY.

(*Continued from page 404.*)

Fracture of the internal condyle (internal oblique fracture) is considered by Samuel Lloyd[21] to be more frequent than fracture of the external condyle in children, and often accompanied by dislocation of the radius, while the ulna follows the displacement of the articular surface of the humerus. Sometimes the fragment is simply split, and remains in its normal relation to the shaft of the bone; at other times it is displaced inwards, upwards, and backwards, and less often forwards and downwards. Occasionally the condyle is completely split away and turned out from the joint. It is often turned forwards, so as to cause an obstruction to the free motion of the forearm or the arm. The fracture invading the joint, and especially as it crosses the humeral fossæ, is very apt to result in some loss of function. The filling-up of these fossæ with callus, next to the displacement of the fragment, is the most serious condition. It can be prevented only by an accurate replacing of the fragment and the gradual change of position of the elbow, so that flexion and extension of he elbow within the limits of safety, and without allowing any rocking of the fragment, becomes very important. If it is impossible to get the fragment back into place, it is better to cut down upon it at once and remove it, rather than to allow it to become fixed in a position that will not allow of future free use of the arm. Lloyd believes that fracture of the internal epicondyle is always extra-capsular, and not likely to be followed by any limitation of motion. He recommends treatment by flexion of the forearm at about a right angle.

E. R. Corson[22] contributes a valuable paper on the mechanism of fracture of the neck of the radius. He believes that it occurs through strain of the orbicular and external lateral ligaments. For instance, in dislocation backwards of the ulna the head of the radius is simply torn off from the neck through the strain on the orbicular ligament, the ulna being forced away from the radius while the radius is fixed against the capitellum of the humerus. The *x* rays have brought to light many instances of this injury; and Corson gives an excellent picture which shows plainly a transverse fracture of the neck, just below the head, which is comminuted, though the ring shadow of the superior articular surface wall indicates that this part is unbroken. This skiagraph gives strongly the impression that the head has been torn off from the neck in the backward dislocation of the ulna. During the act of dislocation the fragments have been displaced forwards.

E. W. H. Shenton[23] also says that fracture of the head of the radius is not always diagnosed without the help of the *x* rays; and, indeed, is not often suspected. He gives a figure in which an impacted fracture at the neck of the radius is seen. Such a condition was suggested before the *x* rays were used; the skiagram only gave confirmatory evidence.

In an experience of three hundred cases of fracture of the lower end of the radius (Colles's), according to M. W. Wace[24] the characteristic "silver fork" deformity was encountered in less than 10 per cent. of the cases, and for the establishment of the diagnosis in all these instances reliance was placed on a far more logical and anatomical sign. This sign is mentioned in Stimson's recent treatise on Fractures or Dislocations. If the surgeon marks the positions of the styloid process of the radius and ulna by pressing the end of the finger into the side of the joint below and against the end of each, he will see that the styloid process of the radius has risen, so that instead of being a quarter of an inch lower (nearer the hand) than that of the ulna, as it usually is, it has risen to the same level, or even above it. Nowhere in German literature is cognisance taken of this phenomenon, whereas in France it has been frequently alluded to, and is known as the "Signe de Langier." It is of the greatest importance, and suffices of itself to establish the nature of the lesion. In estimating this sign the normal wrist must always be taken in comparison. This sign antedates the use of the *x* rays; in addition, it has stood the test of them, and so conspicuously does this altered relation of the styloids figure in skiagrams, that just herein lies the greatest confirmation of its practical use.

As the result of an exhaustive investigation of Röntgen photographs, and dissections and unsewn specimens, J. Lynn Thomas[25] has found that the ulnar styloid process is fractured in from 50 to 60 per cent. of Colles's fractures (143 out of 264 cases), but it is often broken in reversed Colles's fractures; that it may also be fractured in other conditions, viz., fracture of the radius alone or of both bones of the forearm above the site of Colles's fracture, separation of the lower radial epiphysis, and dislocation of the wrist, and also that it may occur alone.

Sir William Stokes,[26] in a clinical note on a case of fracture of the carpal scaphoid, says that, as a proof of how this injury, which he believes occurs much more frequently than has up to this been believed—a view in which Stimson concurs—has been left unnoticed, is the fact that in few of the systematic treatises on fractures, and in none of the older ones, is any mention made of it. In Dublin, the only two cases of this fracture that have been noted have been observed by Bennett. In making the diagnosis, reliance cannot be placed on either symptoms or external physical signs—pain, swelling, crepitation, loss of power, may all be present in the absence of any carpal fracture; and in the absence of Röntgen ray photography no accurate diagnosis can possibly be made as to the nature or exact situation of the lesion producing these signs and symptoms.

G. F. Beatson[27] records a case of the so-called "stave of thumb," or Bennett's fracture, and calls attention to the lack of its general recognition. Not only does this fracture of the base of the metacarpal bone somewhat simulate a dislocation in the living subject, but the pain and swelling that accompany it so mask crepitus that the injury is generally regarded as a "sprain of the thumb." It is true that, even if untreated, the fracture unites with a trivial deformity after all, but at the same time a hand so injured "remains under the best of treatment long disabled, and without treatment for a greatly longer time" (Bennett). In his case, even after the lapse of 16 days, Beatson could still detect some crepitus, and an $x$ ray photograph confirmed the existence of the fracture of the base of the bone. The figure given by him shows that the fracture there depicted is exactly that described by Bennett. It is oblique, detaching the palmar half or more of the articular surface of the metacarpal bone facing the trapezium; while, in addition, the entire metacarpal bone, except the little piece so separated, has slipped backwards, no doubt through the action of the extensor ossis metacarpi, and thus simulates a sublaxation of the bone in this direction. Both these points are clearly brought out in the skiagraph.

**Lower Extremity.**—The fact that 48 cases of depression of the neck of the femur, giving rise to coxa vara, have come under the observation of Royal Whitman[28] within comparatively few years, would seem to indicate that this deformity, from one cause or another, is more common than is generally believed. He places on record another case of separation of the epiphysis in adolescence, an accident that has been demonstrated by Sprengel, and points out the essential differences between this class of cases and those of true fracture of the neck of the femur. He again calls attention to the fact that depression of the neck of the femur, whether it be simple or traumatic, predisposes to progressive deformity. For this reason operative treatment may be indicated at an early stage of the affection as a preventive measure.

As to the technique of suturing the patella after fracture, C. G. Cumston[29] says that the incision should be made so as to give plenty of room and freely expose the joint, especially when the fracture is of long standing. The resulting cutaneous cicatrix should be as distant as possible from the cicatrix of the bone. To fulfil these requirements he makes a horseshoe-shaped incision, keeping at least four or five centimetres away from the borders of the patella. It is commenced above the internal angle of the upper fragment and carried outwards, then brought down almost vertically along the external aspect of the knee, and when below the lower fragment it is carried nearly to the anterior tuberosity of the tibia. To suture the bone strong silver wire appears to him the only satisfactory material to use, and by drilling a triangle in each fragment, with the apices directed towards each other, the wire may be entirely buried in the bone, excepting the point where the ends come out and are twisted. This manner of suturing will give the greatest amount of solidity, and has been entirely satisfactory. When one of the fragments is small and the other large this method cannot be applied; the wire should then be passed through the patellar ligament or through the triceps tendon.

A rare case of complete dislocation of the head of the tibia backwards, upwards, and inwards, and of the patella upwards and outwards is related by E. A. Mills-Roberts.[30] A slate miner, aged 55, was injured when at work in a quarry, his body being violently thrown forwards whilst his leg and foot were fixed. The limb was found to be extended with an appearance of being bent forwards. The skin was uninjured and tensely stretched over the condylar end of the femur, the whole of the articular surface being quite distinct. The absence of the patella from its normal position was perfectly evident to the naked eye. The rounded shape of the condyles was very distinct. The skin below the femur appeared in well-marked folds and wrinkles. The foot was everted. About four inches above and behind the condyles, on the posterior aspect of the thigh, was a well-marked swelling, in which the head of the tibia could be made out. The patella was easily recognised displaced outwards and upwards. The dislocation was easily reduced under an anæsthetic, but there was a little difficulty in reducing the patella. A long posterior splint was applied for three weeks, when gentle passive movement and massage were commenced. Ultimately the patient had full flexion and extension of the knee, and, wearing an Arnold's knee apparatus, was able to follow his employment as miner without any inconvenience.

L. W. Steinbach[31] describes four cases of fracture of the leg in which he employed fixation plates. He applies a silver plate in the form of a cleat to the flat subcutaneous surface of the tibia, secured by small galvanised steel screws. The method is simple, does not complicate treatment, is capable of maintaining the fragments in unyielding opposition, and thereby shortens the time required for bony union. Unimpaired function results without shortening. The plate, which is designed to meet the indications of any fracture of the shaft of the tibia, transverse or oblique, single or multiple, is made of silver $\frac{1}{16}$ in. thick, $3\frac{1}{2}$ in. in length, and $\frac{3}{4}$ in. wide, with perforations for the screws $\frac{1}{2}$ in. apart. An incision slightly exceeding the length of the plate is made through the skin along the median line of the inner surface of the tibia, its centre corresponding to the line of fracture when the bone is broken in one place only, and corresponding to the centre of an additional fragment. The skin is loosened laterally, the periosteum not being disturbed. After removal of serrations, spicules of bone, blood clots, soft tissue, and other interposed or extraneous substances, the fragments are moulded into position, the plate adjusted, and the drill applied through such of the perforations as are selected for the reception of the screws. The drill, slightly smaller than the diameter of the screws to be employed, is carried through the compact tissue into the medullary cavity. Two screws are usually employed at each end, and are secured by means of an ordinary screwdriver. Considerable force is occasionally required to overcome the tendency to displacement of one of the fragments, and has to be applied by the hands of an assistant. The plate seems to be a harmless tenant on the leg, and is permitted to remain in position until bony union has taken place. Its removal is accomplished with the aid of local anæsthesia alone. The method appears to be especially applicable to cases where there is comminution or great displacement.

[21] The Post Graduate, Feb., 1900, p. 134. [22] Annals of Surgery, Dec., 1899, p. 691. [23] The Physician and Surgeon, Mar. 22, 1900, p. 299. [24] New York Med. Record, Mar. 31, 1900, p. 540. [25] Med. Chron., 1900, p. 44. [26] Brit. Med. Jour., May 5, 1900, p. 1075. [27] Ibid., May 5, 1900, p. 1067. [28] Annals of Surgery, Feb. 1900, p. 145. [29] Annals of Surgery, Mar., 1900, p. 310. [30] Lancet, Feb. 3, 1900, p. 308. [31] Annals of Surgery, April, 1900, p. 436.

